# Proteomics and Nucleotide Profiling as Tools for Biomarker and Drug Target Discovery

**DOI:** 10.3390/ijms222011031

**Published:** 2021-10-13

**Authors:** Bent Honoré, Gregory Edward Rice, Henrik Vorum

**Affiliations:** 1 Department of Biomedicine, Aarhus University, Aarhus, DK-8000 Aarhus C, Denmark; 2 Department of Clinical Medicine, Aalborg University, Aalborg, DK-9000 Aalborg, Denmark; henrik.vorum@rn.dk; 3 Exosome Biology Laboratory, Centre for Clinical Diagnostics, University of Queensland Centre for Clinical Research, Royal Brisbane and Women’s Hospital, Brisbane, QLD 4029, Australia; g.rice@uq.edu.au; 4 Department of Ophthalmology, Aalborg University Hospital, DK-9000 Aalborg, Denmark

Proteomics has gone through tremendous development during recent decades. Protein-coding RNA possesses the information to encode the proteins, and, more recently, non-coding RNA has been shown to be an important regulator of cell function and biomarker of pathology and has been used as a putative clinical intervention. In this Special Issue entitled: “Proteomics and Nucleotide Profiling as Tools for Biomarker and Drug Target Discovery” of the *International Journal of Molecular Sciences*, we have collected a review and original articles wherein the authors address these topics. It is apparent that proteomics and nucleotide profiling possess fundamental strengths due to their ability to solve important research issues via a broad approach. The studies presented in this Special Issue cover a variety of diseases, from brain tumours [1,2] to colorectal cancer (CRC) [3,4], thyroid cancer [5], heart failure [6] and renal failure treated with transplantation [7]. Several different platforms are used, from microarrays [6] and antibody arrays [1] to gel-based proteomics using two-dimensional polyacrylamide gel electrophoresis (2D-PAGE) with mass spectrometry (MS) protein identification [3,4], strategies using matrix-assisted laser desorption ionisation time-of-flight mass spectrometry (MALDI-TOF MS) for protein identification [4] and imaging [5] and liquid chromatography–tandem mass spectrometry (LC-MS/MS) with either data-dependent acquisition (DDA) [1,3,6,7,8] or data-independent acquisition (DIA) using sequential window acquisition of all theoretical fragment ion spectra (SWATH) technology [2]. Quantification strategies include label-free quantification [2,3,6,8] as well as labelling with tandem mass tags (TMT) [7] and isobaric tags for relative and absolute quantification (iTRAQ) [1]. The material analysed varies from cultured cell lines [1,3] to tissue biopsies [3,4], formalin-fixed paraffin-embedded (FFPE) tissue [5,7], extracellular vesicles (EVs) [2] and plasma [4,8]. 

First, Dhar et al. [9] reviewed challenges using model (non-human) species to understand disease processes. The proteome within human health is fairly well-established; however, when it comes to the proteomics of some non-human species used as models for disease processes, there is still a long way to go. Dhar et al. [9] reviewed the field by focusing on antibodies, nanobodies and aptamers and asked the following question: among these, which are best for deciphering the proteome of non-model species? Antibodies, especially those that are monoclonal, have been used for some 40 years with great success, but due to their species specificity, they are often not appropriate when other non-model species are investigated. Zebrafish is now a popular model organism with which important discoveries have been made to understand developmental processes, disease progression and therapeutics. However, appropriate antibodies are often lacking. Even when using animal models, such as mice and pigs, for the study of diseases, these may not be the best models to use due to differences in protein expression, where, surprisingly, *Xenopus* may be a better model system. This underlines the necessity to establish more appropriate ways of discovering affinity molecules for such studies. It may now be time to investigate the benefits offered by alternative affinity reagents, such as single-chain antibodies devoid of light chains, i.e., nanobodies, and single-stranded DNA or RNA sequences, i.e., aptamers. A goal of researchers in this field is to develop affinity reagents with cross-species specificity. 

In their experimental sections, two studies addressed the use of biomarkers to diagnose CRC [3,4]. Ludvigsen et al. [3] used a number of strategies to approach diagnostic biomarkers for CRC. Using the advantage of a model system, consisting of cell cultures that include a normal derived colon mucosa cell line and two different colon carcinoma cell lines, putative markers were identified with top-down proteomics using 2D-PAGE isoelectric focusing (IEF) with MS identification of differentially expressed protein spots as well as a direct MS-based bottom-up approach using LC-MS/MS. Putative biomarkers were further analysed in tissue from ten CRC patients via Western blotting with the use of appropriate antibodies. Putative markers included reticulocalbin, calumenin, S100A6 and protein SET. It was emphasised that these proteins need to be further verified in a larger cohort of patients in order to reveal the clinical relevance of the putative markers. The study includes a discussion of the apparent discrepancy that may be obtained when proteins consisting of different proteoforms are analysed by 2D-PAGE and 1D Western blotting. Different isoelectric variants may be detected as differentially expressed by 2D-PAGE, although the protein identified using 1D Western blotting may appear as unchanged due to the combined detection of the variants with 1D Western blotting, as illustrated in the case of triosephosphate isomerase [3]. In a separate analysis, Thorsen et al. [4] performed an impressive proteomic study on 128 CRC tumours from patients with CRC, making comparisons with site-matched normal tissue biopsies to unravel significantly upregulated proteins in tumours with the aim of identifying proteins that may have leaked from the tumour to plasma, where they then may be measured for the early detection of CRC. The authors used a top-down proteomic strategy consisting of 2D-PAGE, nonequilibrium pH gel electrophoresis (NEPHGE) and IEF with subsequent MS protein identification to identify 63 potential serologic biomarkers for the early detection of CRC. In-gel tryptic digestion and protein identification using MALDI-TOF were used. Fluorescence immunohistochemistry was used to confirm the significant upregulation of 10 selected proteins in tumours as compared with non-malignant tissue. Of these, they tested 7 proteins in human plasma from 70 healthy individuals, 70 adenomas, 70 CRC patients and 70 patients with non-cancer disease using a proximity extension assay. Interestingly, one protein, tropomyosin 3 (Tpm3), could significantly discriminate CRC from the other groups, suggesting that this protein could be used as a plasma biomarker in the early detection of CRC.

Two studies focused on brain tumours [1,2]. Gliomas are responsible for more than 60% of all brain tumours. The prognosis, however, is poor. The treatment of choice is surgery, radiotherapy and chemotherapy with temozolomide, giving a median survival amounting to about 15 months. Some patients may not respond to chemotherapy, and, therefore, there is an urgent need to discover new anti-glioma compounds. Izumi et al. [1] used a number of methods to analyse the potential of six low-molecular-weight sesquiterpene lactones isolated from the Brazilian plant *Eremanthus* species for their effect on glioblastoma multiforme (GBM) cell cultures. Of the six tested compounds, two of them, goyazensolide and lychnofolide, reduced cell viability and could pass the blood–brain barrier. Then, the authors used a membrane-based antibody array for 35 apoptosis-related proteins and 26 cell stress-related proteins and concluded that temozolomide preferentially induces apoptosis, whereas goyazensolide and lychnofolide prevent cell proliferation, probably by increasing p27 expression. The two GBM cell lines were treated with the two compounds together with temozolomide and two substances, thapsigargin and tunicamycin, known to induce the unfolded protein response with endoplasmic reticulum stress. Labelling of the tryptic-digested proteins with iTRAQ and subsequent proteomic analyses revealed differences among the two cell lines tested as well as among the different compounds used. No specific enriched pathway or molecular function could contribute to elucidating the specific mechanisms involved in the action of the lactones. A study conducted by Hallal et al. [2] focused on the challenges of the clinical surveillance of patients with GBM. In order to monitor tumour activity, Hallal et al. [2] studied EVs from plasma using SWATH-MS to profile blood EVs. They analysed plasma EVs from 41 pre-operative glioma grade II–IV patients and 11 controls, used the data for alignment with a custom 8662-protein library and were able to measure 4054 proteins in plasma EVs. With this technique, they identified putative circulating EV markers. Principal component analysis showed profile clustering according to glioma histological subtype and grade, and plasma EVs resampled from patients with recurrent tumour progression grouped with more aggressive glioma samples. The authors thereby achieved a most comprehensive and in-depth proteomic coverage of plasma EVs, which can be used as a valuable platform for future biomarker discovery in larger cohorts of patients.

End-stage renal disease is treated with renal transplantation. Survival of the allograft, however, is limited by the development of interstitial fibrosis and tubular atrophy. In order to identify renal biomarkers for this, Mortensen et al. [7] used LC-MS/MS and 10-plex TMT labelling on FFPE tissue from 31 renal transplant patients. Amongst 2687 proteins analysed, they found four proteins strongly correlated with the degree of fibrosis, coagulation factor XIII A chain, uridine phosphorylase 1, actin-related protein 2/3 subunit 2 and cytochrome C oxidase assembly factor 6 homolog, which were highly associated with the degree of interstitial fibrosis. Three of the proteins were also shown to be strongly predictive. Proteins that were negatively correlated with fibrosis were primarily related to metabolism and respiration, while positively correlated proteins were primarily related to catabolic processes, cytoskeleton organisation and the immune response. Thus, cytoskeleton organisation and immune responses are major processes related to renal allograft fibrosis.

Heart failure with preserved ejection fraction (HFpEF) is a complex disease with a lack of successful treatment. Zhang et al. [6] used a rat model to investigate the multi-omics changes in the molecular networks in cardiomyocytes associated with HFpEF. The model is based on Dahl salt-sensitive rats that develop HFpEF when exposed to a high-salt diet for 7 weeks. The group of rats with HFpEF showed significant differences as compared with the control group based on several analyses, namely, shotgun proteomics, microarray analysis, immunohistochemistry, Western blotting and quantitative RT-PCR. Western blot validation was performed on the upregulated proteins Mff, Itga9 and the downregulated Map1lc3a. The multi-omics strategy revealed that multiple pathways were associated with the disease and provided potential targets for the treatment of HFpEF. Inflammatory response and mitochondrial fission were prevailing biological processes that may deteriorate myocyte stiffening.

Proteomic analysis of blood plasma or serum is generally problematic due to the presence of a few proteins, including albumin, immunoglobulins and complement factors accounting for a large majority of the protein content of plasma. A common method to circumvent this in proteomic analyses is the use of immunodepletion of the most abundant proteins prior to analysis. Another method is the enrichment of low-abundant proteins based on affinity capturing using a hexapeptide library with limited capacity for high-abundant proteins prior to analysis. Palstrøm et al. [8] addressed this problem by using four small-molecule affinity-based probes, agarose-immobilised benzamidine (ABA), O-phospho-l-tyrosine (pTYR), 8-amino-hexyl-cAMP (cAMP) and 8-amino-hexyl-ATP (ATP), to remove high-abundant proteins and compared this method with the use of the Multi Affinity Removal System Human 14 (MARS14) and the ProteoMiner protein equalisation method. They found that the ABA-based affinity probe and the ProteoMiner protein equalisation method performed better than all other analysed methods in terms of the number of analysed proteins. Generally, small-molecule affinity-based probes are excellent alternatives to the immune-depletion methods used in studies with the aim of discovering proteomic biomarkers in plasma.

Traditional histopathologic examination of thyroid tumours may in some cases be ambiguous. In order to improve diagnostic classification, Kurcyk et al. [5] combined mass spectrometry imaging (MSI) with a number of different computation approaches to classify thyroid tumours based on tryptic peptide profiles. MSI components showing the most significant differences between different types of tissues were further identified by subjecting corresponding tissue lysates to LC-MS/MS. The authors generally found high accuracy of sample classification. Models based on individual spectra (the single-pixel approach) outperformed the model based on the mean spectra of tissue cores. The authors thereby confirmed the high feasibility of MSI-based approaches to define cancer types based on individual spectra, overcoming the small amounts of heterogeneous material limiting the applicability of classical proteomics.

Overall, the nine contributions to this Special Issue demonstrate that proteomic techniques have considerably developed and improved in recent years. Proteomics is a powerful tool that has brought to attention novel biomarkers, some of which may also be treatment targets. We deeply thank the authors for their valuable contributions to this Special Issue.
